# Maternal Nicotine Exposure Alters Hippocampal Microglia Polarization and Promotes Anti-inflammatory Signaling in Juvenile Offspring in Mice

**DOI:** 10.3389/fphar.2021.661304

**Published:** 2021-05-11

**Authors:** Li Zhou, Xinrong Tao, Gang Pang, Min Mu, Qixian Sun, Fei Liu, Yuting Hu, Huihui Tao, Bing Li, Keyi Xu

**Affiliations:** ^1^ Center for Medical Research, School of Medicine, Anhui University of Science and Technology, Huainan, China; ^2^ Key Laboratory of Industrial Dust Control and Occupational Health, Ministry of Education, Anhui University of Science and Technology, Huainan, China; ^3^ Key Laboratory of Industrial Dust Deep Reduction and Occupational Health and Safety, Anhui Higher Education Institutes, Anhui University of Science and Technology, Huainan, China; ^4^ Engineering Laboratory of Occupational Safety and Health, Anhui Province, Anhui University of Science and Technology, Huainan, China; ^5^ College of Basic Medical Science, Anhui Medical University, Hefei, China

**Keywords:** maternal nicotine exposure, hippocampus, microglia, mouse, Offspring

## Abstract

Accumulating evidence reveal that maternal smoking or perinatal nicotine replacement therapy impairs hippocampal neurogenesis, neural development, and cognitive behaviors in the offspring. Microglia is a source of non-neural regulation of neuronal development and postnatal neurogenesis. In this study, we explored the impact of nicotine on the microglia during the development of hippocampus. Developmental nicotine exposure in a mouse model was conducted by supplementing nicotine in the drinking water to mother mice during gestation and lactation period. We found that juvenile offspring with maternal nicotine exposure presented physical and neurobehavioral development delay and an increase in anxiety-like behavior in the open field test on postnatal day (PND) 20. To further detect possible developmental neurotoxic effects of nicotine in offspring and underlying mechanism, whole genome microarray analysis of the expression profile of the hippocampus was performed on postnatal day 20. Significant alterations in the expression of genes related to inflammatory, neurotransmitter, and synapsis were observed in the hippocampus after maternal nicotine exposure, as compared to the vehicle control. Concurrently, an increase in microglial markers and the presence of M2 polarity state in the hippocampus of the nicotine offspring were observed by histological analysis and confocal z-stacking scanning. The M2 microglial polarization state was further confirmed with *in vitro* primary microglia culture by cytokine array, and double-positive expression of BDNF/Iba1 in microglia by immunohistochemical staining in the juvenile offspring hippocampus was visualized. We also found that nicotine offspring showed an increase of neurite length in the molecular layer and CA1 by Tuj1 staining, as well as an increase in the expression of synapse associated protein, PSD95, but the expression of NeuroD1 in CA1 and CA3 reduced. In summary, maternal nicotine exposure dysregulates immune-related genes expression by skewing the polarity of M2 microglia in the hippocampus, which may cause abnormal cognitive and behavioral performance in the offspring.

## Introduction

Tobacco smoking, nicotine replacement therapy (NRT), e-cigarettes, as well as other nicotine exposure remain a major public health issue across the world (Guerby et al., 2020), and the trend of nicotine usage and exposure continues increasing over the past decades (Dai and Leventhal, 2019; Kapaya et al., 2019). Recent epidemiological studies have shown that 13.8% of pregnant women and 20.1% of non-pregnant reproductive age women smoke (Lopez et al., 2018), and female smoker population has been reported to be more difficult to quit smoking when compared with male smokes (Leventhal et al., 2007). NRT is used to reduce the nicotine withdrawal symptoms experienced by women before, during, and after pregnancy.

Some studies show that NRT is a safer alternative to tobacco smoking for pregnant women despite the lack of information on the toxicological effects of nicotine on the developing fetus (Mark et al., 2015; Mead et al., 2019). Nicotine in lactate can be absorbed by offspring through placenta and lactate and causes behavioral irritation and anxiety in the offspring (Karimiankakolaki et al., 2019). Nicotine exposure during pregnancy is associated with numerous fetal consequences including pre-mature birth, low birth weight, and Sudden Infant Death Syndrome (Salihu and Wilson, 2007; Knopik et al., 2016b; King et al., 2018; Buck et al., 2019), as well as neurodevelopmental disorders, such as attention deficit hyperactivity disorder, autism, and schizophrenia in developing children (Knopik et al., 2016a; Niemela et al., 2016; Golding et al., 2017; He et al., 2017; Quinn et al., 2017; Huang et al., 2018; Marceau et al., 2018). These observations demonstrate that the offspring are vulnerable to nicotine. Therefore, understanding the unique effects of nicotine use on offspring is critical for treating and preventing the adverse consequences that follow.

Previous studies have indicated that the effects of maternal nicotine exposure on offspring are intricated. Being altricial species, both humans and rodents undergo a pre and postnatal series of highly regulated, sequential changes to cell specialization, known as critical periods in central nervous system (CNS) development (Morgane et al., 1993). Exposure to nicotine, particularly when occurs during critical developmental periods, can adversely affect brain, immune system (Yan et al., 2020), and other systems throughout the body (Chen et al., 2020; Jamshed et al., 2020; Jian et al., 2020; McAlinden et al., 2020; Miranda et al., 2020). Chronic nicotine exposure *in utero* could alter neuronal cytoarchitecture, nicotinic acetylcholine receptor (nAChR) expression, and function of neurotransmitter systems (Lv et al., 2008; Gold et al., 2009). Activation of nAChRs by nicotine could induce developmental problems in premature stage, such as cholinergic-mediated signaling, which initiates neuronal transformation from replication to differentiation (Smith et al., 2010). Further, activation of nAChRs by nicotine impairs the development of neurotransmitter systems, especially dopamine (DA) (Azam et al., 2007; Zhang et al., 2020), norepinephrine (NE) (Sterley et al., 2014), and serotonin (5-HT) (Lee et al., 2018). In addition to alternations in nAChR expression and neurotransmitter system function, a recent study suggests that nicotine exposure during early development stage alters neurotrophins and neuroinflammation (Zelikoff et al., 2018a).

During early stage of postnatal development, the CNS is sensitive to external stimuli and internal neurotrophins, including nerve growth factor (NGF) and brain-derived neurotrophic factor (BDNF), which modulate brain plasticity to adapt to the environment (Berry et al., 2012). Several studies have reported the effects of maternal nicotine exposure on BDNF levels in different brain areas (Buck et al., 2019). Besides neurons, BDNF receptor is also expressed in M2 microglia (Ding et al., 2020). To date, only a paucity of research explored the relationship between nicotine and BDNF, and no study has yet elucidated the exact effect of maternal nicotine exposure on microglia BDNF of juvenile offspring.

Microglia are the resident innate immune cells of the CNS and exert functions of host defense and maintenance of normal tissue homeostasis, along with the support of neuronal development and processes in the healthy brain. The abnormality of microglial activation plays a critical role in neurodegenerative disease (Maleszewska et al., 2020; Nasi et al., 2020). Inflammatory mediators influence the brain during development. Neurodevelopmental disorders such as autism spectrum disorders, cognitive impairment, cerebral palsy, epilepsy, and schizophrenia are associated with early inflammation (Jiang et al., 2018). Nicotine has been shown to directly and indirectly mediated neuroinflammation (Sallam et al., 2019; Han et al., 2020), which can lead to neurodevelopmental disorders and lasting effects. However, activation of microglia during development due to nicotine exposure could have profound developmental consequences. Nicotine attenuated neuronal loss and decreased the expression of microglial activation markers of the hippocampal CA1 region in the eclampsia-like rat, which improved fetal outcomes (Li et al., 2016). Nicotine reduced neurogenesis and altered microglial profiles in the hippocampal DG (dentate gyrus) in an early postnatal period (Nakayama et al., 2019).

This study aimed to assess the *in vivo* effect of nicotine on neurodevelopment in juvenile offspring exposed to nicotine during critical early life stages. We identified the signature of mRNA in the hippocampus of offspring after maternal nicotine exposure to evaluate the hippocampal transcriptome alterations. The expression of inflammatory genes, as well as neurotransmitters and synaptic-associated genes, were changed. In this study, we for the first time reported that maternal nicotine exposure promoted M2-like microglial polarization to influence the hippocampal immune microenvironment in the offspring. We also found that maternal nicotine exposure promoted elongation of axons and increased the expression levels of synapse-related proteins (PSD95) in the hippocampus of offspring, but decreased the expression of a single transcription factor, NeuroD1. In conclusion, maternal nicotine exposure promotes the polarization of hippocampal microglia to M2 and alters inflammatory factors in the offspring, which may further modulate the morphology and function of neurons in the hippocampus, leading to developmental defects.

## Materials and Methods

### Animals

Adult C57BL/6 mice were purchased from the Changzhou Cavion Experimental Animal Co, Ltd. (license number SCXY (Su) 20110003). The mice were housed in a room maintained on a standard 12 h light-dark cycle (lights on at 07:00 AM), with constant temperature and humidity (24 ± 2°C and 50%, respectively), and with free access to food and water. All procedures and operations were performed in strict accordance with the guidelines as described in the National Institutes of Health Guide for the Care and Use of Laboratory Animals (NIH Publication No. 8023, revised 1978) and were approved by the Institutional Animal Care and Use Committee at Anhui University of Science and Technology.

### Maternal Nicotine Exposure

Adult C57BL/6 female mice (*n* = 12) were given nicotine (Sigma, N-3876; 200 μg/ml) in drinking water containing 1% saccharin (Shyuanye; 128–44–9) starting from 2 weeks premating until the offspring were weaned. The female control mice (*n* = 9) received drinking water containing 1% saccharin. 2 weeks after drinking nicotine solution, all female mice were paired with male mice at a 3:1 ratio until they gave birth. The offspring of either sex were used on postnatal day 20 (PND 20), but included an equal number of male and female mice and were distributed randomly for subsequent experiments (Supplementary Table 1).

### Physical and Neurobehavioral Development

The birth weight of the offspring was weighed separately, 2∼3 mice were selected at random from each cage for subsequent experiments. Eye opening was based on the degree of binocular opening that was equal to or greater than 1/2 of normal palpebral fissure. The olfactory reflex test started on PND 11 for consecutive days by placing the offspring in the center of a box with a clean absorbent cotton on one side and an odorous absorbent cotton on the other side to evaluate whether the offspring could distinguish the smell of the cage and reach the side of the absorbent cotton with the smell. Auditory startle test started on PND 11 for consecutive days, the offspring were placed in a cage alone and a metal block 15 cm away from the offspring was struck. The positive reactions include body suddenly curl, arch, and two consecutive positive stimulus responses were defined as the standard.

### Open-Field Test

Open-field test was performed at PND 20. Locomotor activity was recorded and analyzed *via* an overhead video camera interfaced with behavioral tracking software EthoVision® XT 5.1 (Noldus Information Technology, Netherlands). The experiment was accomplished in a peaceful environment, and mice were acclimated to the behavioral recording room for 3 days, 60 min a day. Then mice were softly placed in the center of an open-field Plexiglas clear chamber (30 × 30 × 35 cm) and allowed to move freely for 5 min. The zone of the chamber was divided into the peripheral zone (area within 7.5 cm away from the edge) and the central zone (the rest area). Behavior test was carried out between 14:00 and 18:00, and all chambers were cleaned fully with 10% alcohol between trials to remove odor residue. The basal exploration activity was assessed by the total distance traveled (cm) and immobility (s), and anxiety-like behaviors were measured by the duration in the center area.

### Histological Staining

#### Hippocampal Tissue Preparation

After opening the skull and cautious removal of adjacent, non-neural tissue, isolated total hippocampal tissue including dorsal and ventral was collected into a container and cooled on ice, then shift into ice-cold protein extraction buffer for western blot analysis or Trizol reagent for RT-qPCR analysis. The rest mice were deeply anesthetized and perfused transcardially with cold phosphate-buffered saline (PBS) followed by 4% paraformaldehyde (PFA) in 0.1 M PBS. Isolated brains were transferred into 4% paraformaldehyde in PBS (pH 7.4) for histological analysis.

#### Nissl Staining

The brains of mice were dissected and fixed in 4% paraformaldehyde for 24 h. A 4 mm-thick coronal section, including the bilateral hippocampus, was excised from the brains. The sections were fixed in 4% paraformaldehyde for another 24 h, dehydrated in alcohol, cleared with xylene, and embedded in paraffin. The paraffin-embedded brain sections were sliced at 5 μm thickness and Nissl-stained with 1% thionin, then observing the morphology of neural cells and damage of pyramidal neurons in the hippocampus of the two groups. While glia was counted separately in each field; glia cells were readily distinguished from neurons by their size, nuclear shape, cytoplasm, location, and characteristic staining (Roy et al., 2002).

#### Immunohistochemistry Staining

Double-labeling immunohistochemistry and colocalization analysis for microglia-derived BDNF were performed using dual staining of BDNF and Iba1. Paraﬃn-embedded hippocampal sections were prepared as described above. 5 μm-thickness hippocampal slices were stained against BDNF and Iba1. In brief, brain slices were baked at 60°C for 6 h, and the slides were deparaﬃnized in xylene and hydrated in gradient alcohol, and then rinsed in dd-H2O. Antigen retrieval was performed by incubation in 10 mM sodium citrate buﬀer via steam for 30 min. Endogenous peroxidase in the tissue was blocked by incubation with 3% H2O2 in dark for 10 min. Tissues were blocked for 1 h in 3% BSA-containing PBST and then incubated with primary antibodies at 4°C overnight. After rewarming for 40 min, then the sections were rinsed and incubated with secondary antibodies, donkey anti-goat IgG H&L, and donkey anti-rabbit IgG H&L for 40 min at room temperature. Lastly, the slices were stained with AP substrate (Vector Laboratories, SK-5100) for 10 min and then stained with DAB substrate (NJJCBio, W026-1-1) for 10 min and counterstained with hematoxylin. Slices were mounted and covered with a permanent mounting medium (Vector Laboratories, H-5000). Images were acquired with a BX50 microscope (Olympus, Tokyo, Japan).

For Tuj1 staining, slices were incubated with the anti-Tuj1 antibody at 4°C overnight. The second antibody was goat anti-rabbit IgG H&L, and then stained with DAB substrate for 10 min and counterstained with hematoxylin for 30 s. The stained slides were immersed in a graded series of ethanol and then, xylene to dehydration and transparency of tissues. Finally, slices were sealed with neutral gum and acquired images.

#### Immunofluorescence Staining

Harvested brains were further fixed with 4% PFA, soaked in 30% sucrose solution before embedding in OCT media. The hippocampal brains were then cut into 30 μm coronal frozen sections. Every 12th section was collected and processed. Slides were permeabilized with 0.1% Triton X-100 in PBS (PBST). After PBST washing, the slices were blocked with 5% goat serum (Beyotime Biotechnology, C0265) for 1 h before being incubated in anti-Iba1 antibody at 4°C overnight. After PBST washing, slices were incubated with Goat anti-Rabbit IgG H&L for 1 h at room temperature. Nucleus was counterstained with DAPI (1:2000; Beyotime Biotechnology, 1002). Slices were mounted with Antifade Mounting Medium (Beyotime Biotechnology, P0126) and stored at 4°C in dark. Images were acquired with an Olympus FV3000 laser scanning confocal microscope (Olympus, Waltham, United States), and a z-step size of 2 um. Z-stacks ranged from 24 to 26 um in thickness.

For Iba1 and NeuroD1 Double staining, slices were incubated with anti-Iba1 antibody and anti-NeuroD1 antibody at 4°C overnight. The second antibody was Goat anti-mouse IgG H&L and Goat anti-rabbit IgG H&L. Subsequent steps were performed as above. Primary and secondary antibodies were all listed in Table 1.

**TABLE 1:** Antibodies used in this study.

Antigen	Host	Dilution ratio	Company
Iba1	Rabbit	1:500 (IF)	Wako, 019–19,741
Iba1	Rabbit	1:1,000 (WB)	Abcam, ab178846
Iba1	Goat	1:300(IHC)	Novus bio, NB100–1028
BDNF	Rabbit	1:500(WB)	ABclonal, A16299
BDNF	Rabbit	1:300(IHC)	Abcam, ab108319
Tuj1	Rabbit	1:500	Abcam, ab18207
GAPDH	Rabbit	1:10,000	Proteintech, 10494-1-AP
GAD1	Rabbit	1:800	Proteintech, 10408-1-AP
PSD95	Rabbit	1:800	Proteintech, 20665-1-AP
NeuroD1	Mouse	1:500	Abcam, ab60704
Goat anti-rabbit IgG H&L	Goat	1:1,000	Abcam, ab6721
Donkey anti-goat IgG H&L	Donkey	1:1,000	Abcam, ab6885
Donkey anti-rabbit IgG H&L	Donkey	1:1,000	Abcam, ab6803-AP
Goat anti-rabbit IgG H&L	Goat	1:1,000	Life technology, A-11008
Goat anti-mouse IgG H&L	Goat	1:1,000	Life technology, A-28175
Goat anti-rabbit IgG H&L	Goat	1:1,000	Life technology, A-11037

### RNA Isolation, cDNA Synthesis, and Real-Time Quantitative PCR

Total RNA was isolated from the hippocampus using Trizol extraction method according to the manufacturer’s instructions (Invitrogen). For cDNA synthesis, 5x All-In-One RT MasterMix (abm, Jiangsu, China) was used following the manufacturer’s instructions using the PCR instrument (Hema9600). Real-time polymerase chain reaction was next performed on a QuantStudio three Real-Time PCR System (Thermo Fisher Scientific, United States) for the detection of BDNF, CXCL10, JUNB, IL-1β, IL-4, and the endogenous control GAPDH. For one amplification reaction, 5 μl of GoTaq^®^ qPCR Master Mix (Promega, Madison, WI, United States), 0.4 μl of primer (10 μM), 1 μl of cDNA, 0.1 μl of CXR, and 3.5 μl of DNase-free water were mixed for detection. All qPCR primers were designed using Primer3 software and verified using the BLAST-like alignment tool. The oligonucleotides used for qPCR are listed in Table 2. The expression levels of the targeted genes were normalized to that of the endogenous control GAPDH by QuantStudioTM Design&Analysis Software (v1.3.1) based on the 2−ΔΔCt formula.

**TABLE 2:** The primer sequences used for real-time quantitative polymerase chain reaction analyses.

Gene	Forward sequence (5′-3′)	Reverse sequence (3′-5′)
GAPDH	GTG​GGT​GCA​GCG​AAC​TTT​AT	CAC​TGA​GCA​TCT​CCC​TCA​CA	
BDNF	GCC​TTT​GGA​GCC​TCC​TCT​AC	TCA​GTT​GGC​CTT​TGG​ATA​CC	
CXCL10	CCC​ACG​TGT​TGA​GAT​CAT​TG	GAG​GCT​CTC​TGC​TGT​CCA​TC	
CCL12	GGT​ATT​GGC​TGG​ACC​AGA​TG	CAA​GGA​TGA​AGG​TTT​GAG​ACG	
JUNB	ACG​GAG​GGA​GAG​AAA​AGC​TC	AAG​GCT​GTT​CCA​TTT​TCG​TG	
IL-1β	TGG​ACC​TTC​CAG​GAT​GAG​GAC​A	GTT​CAT​CTC​GGA​GCC​TGT​AGT​G	
IL-4	AAC​GAG​GTC​ACA​GGA​GAA​GG	TCT​GCA​GCT​CCA​TGA​GAA​CA	

### Microarray and Computational Analysis

The hippocampal RNA was analyzed using Arraystar RNA Flash Labeling Kit. After RNA labeling and hybridization, slides were scanned by Agilent DNA Microarray Scanner. Raw data were normalized and analyzed using GeneSpring GX v12.1 software (Agilent Technologies, Santa Clara, CA). Differentially expressed mRNA between two groups were identified through *p* value (cut–off: 0.05) and FC (cut–off: 1.5) sifting. Pathway and gene ontology (GO) analysis were applied to determine the roles of these differentially expressed mRNAs on biological pathways or GO terms. Hierarchical clustering and combined analysis were performed using in-house scripts (Kangcheng Biotechnology Company). The microarray data were deposited in Gene Expression Omnibus (GEO accession: GSE166311).

### Primary Microglia Cultures and Cytokine Measurement by Protein Array

#### Primary Microglial Culture

Primary cultures of mice mixed glial cells (microglia and astrocytes) were obtained as described previously (Schildge et al., 2013) with a few modifications. Briefly, whole brain isolates were extracted from neonatal C57BL/6 mice pups aged 2–4 days and transferred into a new dish with 5 ml L-15 solution (Leibowitz L-15 + 0.1% BSA +1% Pen/Strep, Gibco) on ice. Mixed glial cultures were prepared from cerebral cortices. cortices were gently pipetted up and down 10 times and mechanically dissociated before cortical tissues were treated with 0.05% trypsin for 30 min at 37°C. Trypsinization was stopped by 10% fetal bovine serum (FBS). Mixed glia cells in the supernatant were collected and cultured in high glucose DMEM supplemented with 10% FBS, 1% penicillin/streptomycin, and 2 mM of L-glutamine (D-10 medium). Cells were cultured in a humidified incubator at 37°C (5% carbon dioxide). The culture medium was half-replaced every 4–6 days. Mixed glia cells became confluence after 14–21 days in culture medium. Loosely attached primary cultures of microglia were prepared after shaking at 100 rpm (Dragon Lab, #SK-D 1807-E, China) for 2 h at 37°C. The cell pellets were re-suspended and re-plated with D-10 medium for the following experiment.

#### Evaluation of Cytokines Collected From Cell Culture Supernatant

Primary microglia were re-plated into a 12-well plate. Blank served as the unstimulated control for each time point, lipopolysaccharide (LPS)-stimulated primary microglia as the pro-inflammatory control (LPS group), nicotine group, and LPS plus nicotine group were set up in this experiment. Microglia were stimulated with LPS, nicotine, or LPS plus nicotine at the final concentration of 10 μg/ml LPS, 10 μmol nicotine, 10 μg/ml LPS plus 10 μmol nicotine. Microglia cells were incubated with LPS for 24 h in LPS group. LPS plus nicotine group were pretreated with LPS for 30 min before application of nicotine for analyzing anti-inflammatory effects of nicotine. Medium were collected at 12/24 h, respectively. Then, Supernatants were obtained by centrifugation at 1200 rpm for 10 min at 4°C, and stored at −80°C until they were used for cytokine assay. Cytokines in the supernatant samples were detected using Quantibody® Mouse cytokine array 1 (Ray Biotech, United States) according to the manufacturer’s instructions. In brief, the signals (green fluorescence, Cy3 channel, 532 nm excitation, and 542 nm emission) were captured using an InnoScan 300 Microarray Scanner (Innopsys, France). Quantitative data analysis was performed using Ray Biotech mouse Cytokines Array l software (GSM-CYT-1 Q-Analyzer).

### Western Blot

The total protein of hippocampus was homogenized with RIPA lysis buffer (P0013B, Beyotime Biotechnology, Shanghai, China) containing PMSF, and phosphatase inhibitor on ice following incubation for 30 min at 4°C. Hippocampal lysates were centrifuged at 10,000 ×g for 10 min at 4°C. Supernatant was collected and the protein concentration was determined by a Bicinchoninic acid Protein Assay kit (P0009, Beyotime, Shanghai, China). The loading buffer was used to adjust the protein concentration and 40 µg protein was loaded and separated by 10% sodium SDS–PAGE (Beyotime Biotechnology) before being transferred to a PVDF membrane (Millipore). After 1 h BSA blocking, membranes were incubated with rabbit anti-Iba1 antibody, rabbit anti-BDNF antibody, rabbit anti-PSD95 antibody, anti-GAD1 antibody, and rabbit anti-GAPDH. After being incubated with the second antibody Goat Anti-Rabbit IgG H&L (HRP), membranes were washed with TBST 3 min for 5 times. The protein bands were visualization with chemiluminescent HRP substrate (P90720, Millipore Corporation, Burlington, MA) and detected by Molecular Imager ChemiDoc™ XRS + analysis system (BioRad Co., Hercules, CA). ImageJ was used for quantitative Western Blot analysis. All experiments were repeated four times.

## Statistical Analysis

All data were plotted and analyzed using GraphPad Prism (6.01). The results were represented as the mean ± standard error means (SEM). A two-way analysis of variance (ANOVA) for the difference among three or more groups. A two-way repeated measures ANOVA for the difference of body weight were performed using SPSS software package (IBM). The difference among the two groups were analyzed using unpaired Student’s t-tests as appropriate. A probability level of *p* value <0.05 was considered statistically significant.

## Results

### Maternal Nicotine Exposure Altered Physical and Neurobehavioral Development in the Juvenile Offspring

**FIGURE 1:**
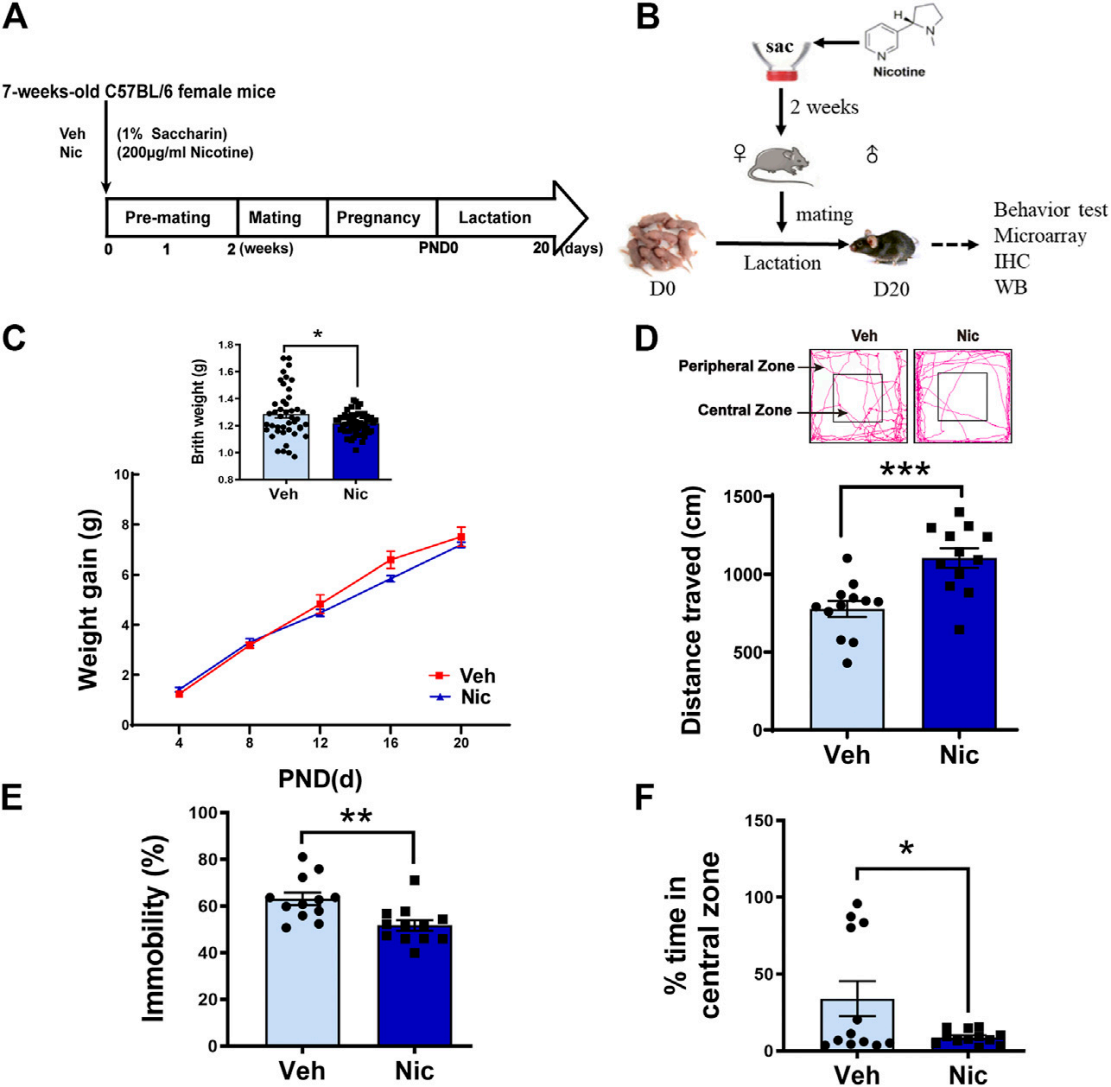
**Maternal nicotine exposure altered the offspring’s physical development and exploration behavior.** The experimental paradigm of maternal nicotine exposure **(A)** and tests in the offspring **(B)** were showed. Mice were terminated on PND20, and brain tissues were collected for further experiments after behavior tests were completed. **(C)** Birth weight and body weight gain throughout development were recorded. Open field test revealed that maternal nicotine exposure significantly increased the total distance traveled **(D)** of the offspring, but visibly reduced the immobility time **(E)**, and exploration time in the center zone **(F)**, as compared to the vehicle control (lower panel). The representative track diagram of open field test was showed (upper panel); *p < 0.05, **p < 0.01, ***p < 0.001, all data are means ± SEM.

Maternal nicotine exposure and tests in the offspring were performed according to the experimental paradigm showed in Figures 1A,B. Birth weight was measured from PND0-20. Maternal nicotine exposure causes low-birth weight (*t* = 2.6, *p* = 0.0107, vehicle: *n* = 44, nicotine: *n* = 60; Figure 1C, upper panel). Ten mice from five litters with similar birth time in each group (*n* = 2 mice/litter) were separately weighted at PND0, 4, 8, 12, 16, and 20. A two-way repeated measures ANOVA on body weight revealed a significant main effect of time (F (4, 36) = 496.523, *p* < 0.001, *n* = 10) and time × group interaction (F (4, 36) = 4.041, *p* = 0.008, *n* = 10), but no difference in group (F (1,9) = 0.626, *p* = 0.449, *n* = 10; Figure 1C, lower panel). The birth weight was significantly reduced in the nicotine group. The physical and neurobehavior development during lactation in the two groups were evaluated every day from PND0-20. Nicotine offspring showed significantly developmental retardation relative to those in vehicle mice: eye-opening (Veh: 12.79 ± 0.11, Nic: 15.17 ± 0.13; *t* = 13.65, *p* < 0.0001, vehicle: *n* = 14, nicotine: *n* = 17), auditory startle (Veh: 11.50 ± 0.20, Nic: 12.75 ± 0.31; *t* = 3.498, *p* = 0.0023, vehicle: *n* = 14, nicotine: *n* = 8), and olfactory reflex (Veh: 11.14 ± 0.10, Nic:12.05 ± 0.16; *t* = 4.65, *p* < 0.0001, vehicle: *n* = 14, nicotine: *n* = 17; Table 3). Open field test revealed that maternal nicotine exposure increased total distance traveled (*t* = 4.018, *p* = 0.0006, *n* = 12; Figure 1D), and significantly reduced the immobility time (*t* = 3.231, *p* = 0.0038, *n* = 12; Figure 1E), and exploration time in the center on PND 20 in offspring, as compared to the vehicle offspring (*t* = 2.198, *p* = 0.0388, *n* = 12; Figure 1F). These data indicate that maternal nicotine exposure leads to weight loss, neurodevelopmental delay, and reduce exploration behavior in their offspring.

**TABLE 3:** Physiological development index in each different group.

Observation	Vehicle (n = 14)	Nicotine (n = 8–17)	Effect
Smell pups (%)			
PND11	85%	18%	
PND12	100%	76%	P < 0.001****
PND13	100%	100%	
Hearing pups (%)			
PND11	64%	0.0	
PND12	86%	50%	*p* = 0.002**
PND13	100%	75%	
PND14	100%	100%	
Eye opening pups (%)			
PND11	0.0	0.0	
PND12	21%	0.0	
PND13	100%	0.0	P < 0.0001****
PND14	100%	5%	
PND15	100%	82%	
PND16	100%	100%	

### Maternal Nicotine Exposure Increased Number of Microglial Cells in the Hippocampus of Juvenile Offspring

**FIGURE 2:**
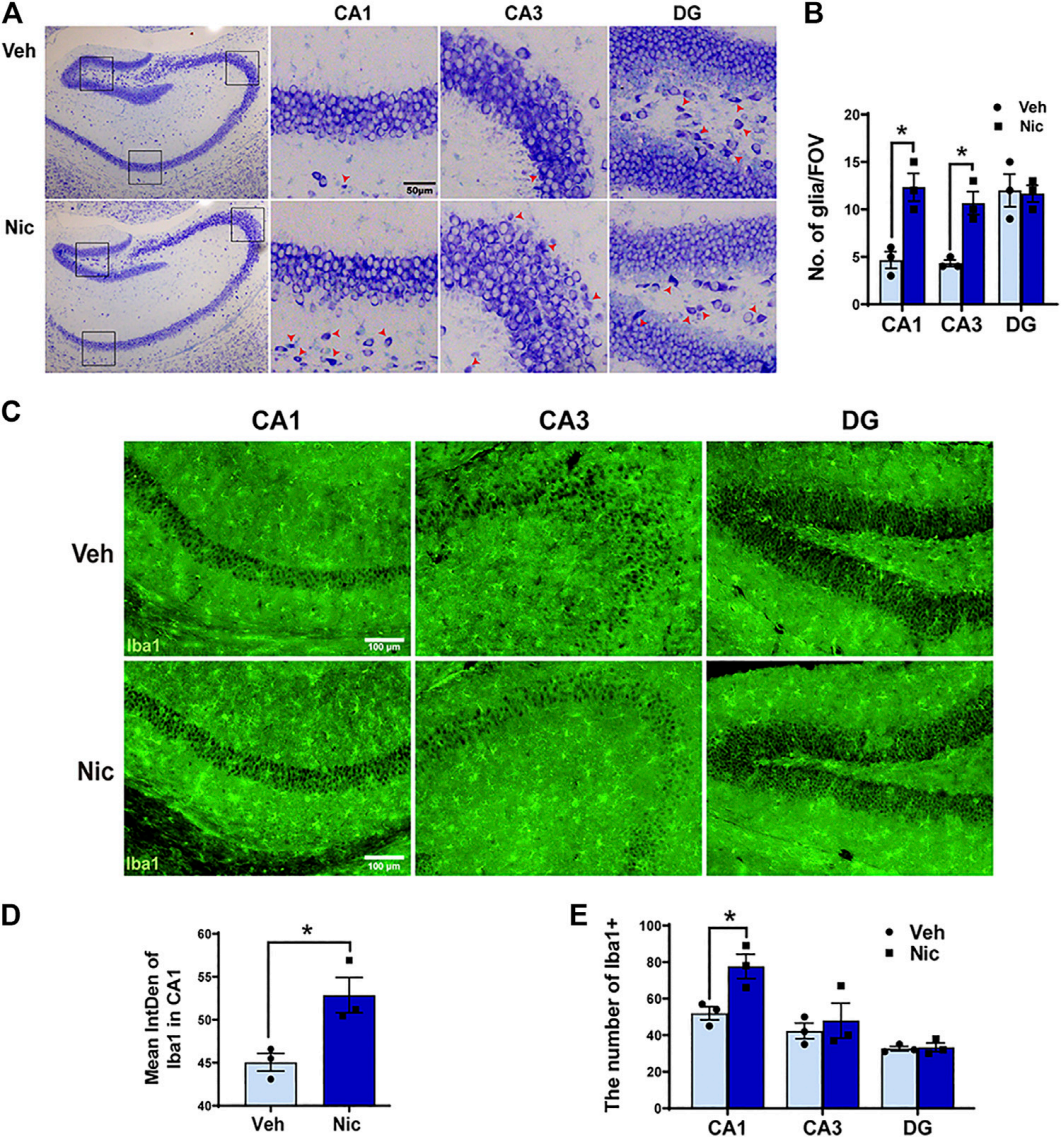
**Maternal nicotine exposure increased the number of neuroglia microglia, but not pyramidal neurons in the hippocampus of pups. (A)** Photomicrograph showed the whole hippocampal sample in the coronal plane (two pictures on the left-most side). DG, CA1, and CA3 subfield are indicated by the black frame in the left-most image. Maternal nicotine exposure increased neuroglia (arrowhead) in DG, CA1, and CA3. **(B)** Neuroglial cells were quantified in the hippocampus. **(C)** Iba1-positive cells in the offspring’ hippocampi were analyzed by immunofluorescence staining using floated section. The hippocampus of 20 day old offspring from the maternal nicotine-exposed group has more microglia cells (green) than the vehicle control. Representative images were shown. The quantification of immunofluorescence staining by ImageJ showed that the expression **(D)** and number **(E)** of Iba1 + cells increased obviously in the nicotine group. Scale bar: 50 μm (A); 100 μm (B); *p < 0.05, all data are means ± SEM.

Nissl staining showed the whole hippocampal sample (low magnification, ×40) in the coronal plane from the vehicle and nicotine group (two pictures on the left-most side). DG, CA1, and CA3 subfield was indicated by the black frame (Figure 2A). Maternal nicotine exposure significantly increased the number of neuroglia (arrowheads) in CA1 (*t* = 4.511, *p* = 0.0107, *n* = 3) and CA3 (*t* = 3.712, *p* = 0.0206, *n* = 3; Figure 2B), but not in DG (*t* = 0.172, *p* = 0.8722, *n* = 3). Additionally, the expression of microglia marker Iba1 in the hippocampus was tested by immunofluorescent staining (Figure 2C). The expression intensity and the number of Iba1 positive cells were analyzed and counted using ImageJ, independently. The fluorescence intensity (*t* = 3.408, *p* = 0.0271, *n* = 3; Figure 2D) and count of Iba1+ cells in CA1 (*t* = 3.396, *p* = 0.0274, *n* = 3; Figure 2E) increased dramatically in juvenile nicotine group, but the number of Iba1+ microglia cells in DG or CA3 was not statistically significant (Figure 2E).

### Microarray Analysis Identified Differentially Expressed Genes in the Hippocampus of the Juvenile Nicotine Offspring

Although the mechanisms of neuron-microglia signaling in regulating brain function *in vivo* have been well studied, the cellular and molecular mechanism of its action in the nicotine-exposed hippocampus during the development is still unclear. To investigate the influence of maternal nicotine exposure on microglia-mediated inflammatory response and neuronal function in the gene expression profile of hippocampus, microarray analysis was performed in 20 days hippocampus. The Agilent Feature Extraction Software was used to identify differentially expressed mRNA in nicotine and vehicle offspring. The threshold of volcano plot filtering used to screen the differentially expressed mRNAs was a fold change ≥1.5.

**FIGURE 3:**
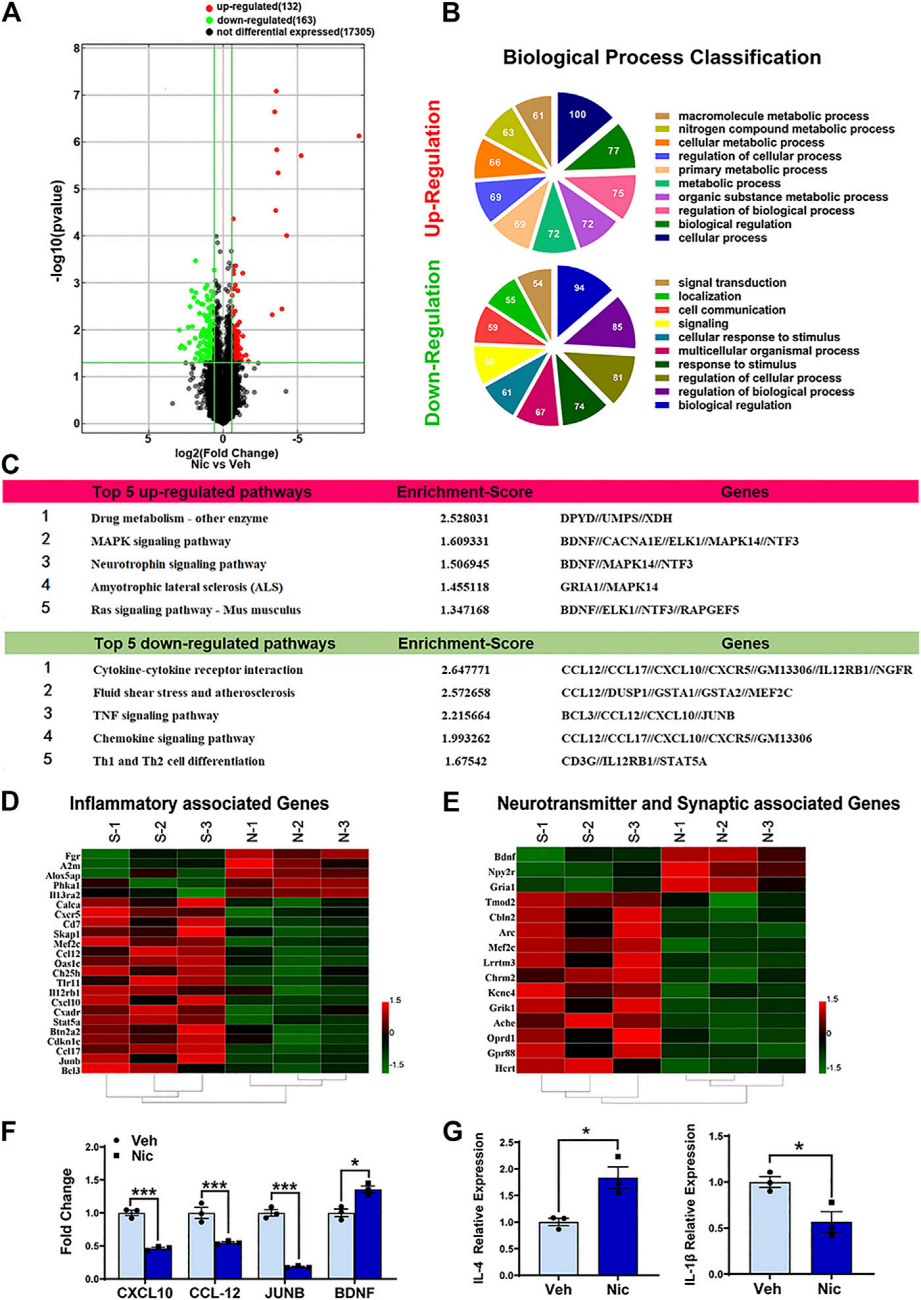
**Bioinformatic analysis of the expression profiles in 20** **day old offspring’ hippocampus. (A)** The distribution of mRNA profiles was illustrated by volcano plots. There are 295 differentially expressed genes. “Red” indicates high relative expression, and “green” indicates low relative expression. **(B)** The pie chart showed the top 10 significant enrichment terms represented by the biological process. Larger sectors represent more significant enrichment. Varieties of biological functions were expressed differently after maternal nicotine exposure in the hippocampus of the PND20 mice, as compared to the vehicle control. **(C)** The top five upregulated and downregulated pathways in which hippocampus differentially expressed genes involved were listed. Hierarchical clustering heatmaps of inflammatory genes **(D)**, neurotransmitter, and synaptic associated genes **(E)** were generated using HEML software. **(F)** Confirmation of the microarray results, the expression of TNF and neurotrophin signaling pathway-related genes were validated by quantitative real-time PCR. **(G)** The representative genes of different inflammatory responses, IL-4 and IL-1β, were detected by quantitative real-time PCR. The columns represent the log-transformed median fold changes in expression relative to the vehicle group. *p < 0.05, all data are means ± SEM.

In the mRNA expression profiling data, 295 differently expressed mRNAs in the hippocampus of the nicotine offspring have been identified (Figure 3A). Compared to vehicle offspring, 132 mRNAs were significantly upregulated, and 163 mRNAs were obviously downregulated in nicotine offspring, respectively. Then we performed ontologic pathway enrichment analysis for the differently expressed genes enriched with attention to GO biological processes. We found that the most enriched GOs targeted by the upregulated and downregulated transcripts were involved in a variety of functions including metabolism, cellular process, biological regulation, signal transduction, cell communication, response to stimulus, and multicellular organismal regulation (Figure 3B).

The KEGG database was used to investigate the pathways in which the differentially expressed genes are involved. The 10 most enriched pathways (top 5 up-regulated and top 5 down-regulated pathways) in KEGG pathway annotations are shown in Figure 3C. Specifically, the upregulated pathways included drug metabolism-other enzyme, MAPK signaling pathway, neurotrophin signaling pathway, amyotrophic lateral sclerosis (ALS), and Ras signaling pathway. The downregulated pathways involved cytokine-cytokine receptor interaction, fluid shear stress and atherosclerosis, TNF signaling pathway, chemokine signaling pathway, and Th1 and Th2 cell differentiation.

To validate the results of the mRNA microarray assay, quantitative RT-PCR analysis was performed. The experiment demonstrated that CXCL10, CCL12, and JUNB, which relate to the TNF signaling pathway, were down-regulated significantly by nicotine. Yet, maternal nicotine exposure upregulates BDNF expression in the juvenile offspring hippocampus. These data were mostly consistent with the results of the microarray analysis.

To gain insight into the inflammatory response modulated by nicotine in the hippocampal microenvironment, the expression levels of IL-4 and IL-1β were examined by quantitative RT-PCR (Figure 3G). Our data indicated that nicotine leaded to an increase in IL-4 (anti-inflammatory cytokine) (*t* = 3.892, *p* = 0.0177, *n* = 3) and a decrease in IL-1β (inflammatory cytokine) (*t* = 3.485, *p* = 0.0252, *n* = 3). These data suggested that maternal nicotine exposure mainly led to anti-inflammatory responses in the hippocampus of juvenile offspring.

### Prenatal Nicotine Exposure Induced Significant Changes in Transcripts for Many Anti-Inflammatory Genes, as well as Those Related to Neurotransmitter and Synaptic-Associated Genes in Offspring

Selected transcripts associated with inflammatory response were shown (Figure 3D). Nicotine exposure significantly increased the expression of inflammatory-associated transcripts Fgr, A2m, Alox5ap, Phka1, and Il13ra2, while decreased expression of the inflammatory chemokine CXCL10, CCL12, CCL17, CXCR5. Selected representative genes, CXCL10, CCL12, JUNB, BDNF, were confirmed with RT-qPCR (Figure 3F). Taken together, these data show that nicotine exposure has contrasting effects on immune-associated transcripts. Transcripts associated with synaptic and neurotransmitter function were also shown (Figure 3E). Nicotine dramatically suppressed the expression of transient gene Arc in the juvenile offspring, which is implicated in several types of synaptic plasticity, including synaptic scaling, long-term potentiation, and long-term depression. On the other hand, nicotine significantly increased the transient gene BDNF. BDNF protein plays a critical role for the survival of neural cells after brain injury.

### Nicotine Suppresses LPS-Induced Release of Inflammatory Cytokines in Primary Microglia Cells

**FIGURE 4:**
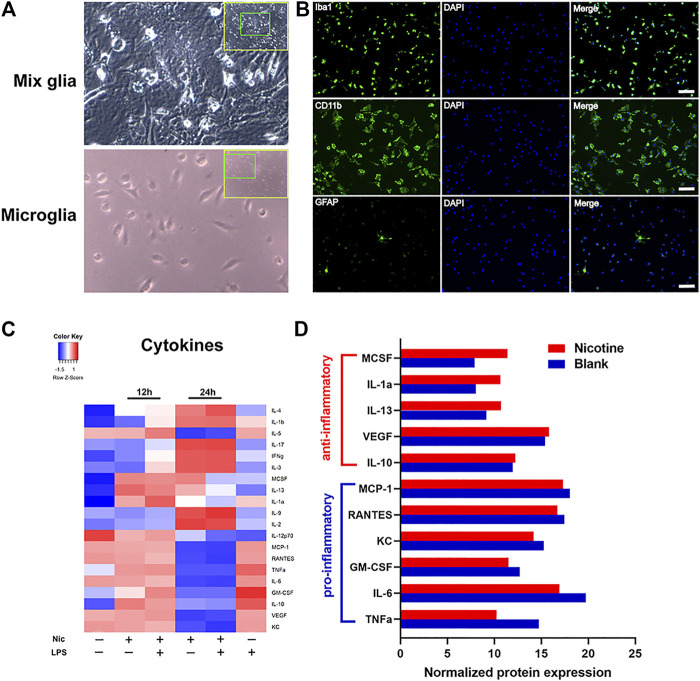
**Nicotine exposure suppressed inflammatory cytokine revealed by protein array.** Primary microglia were isolated from postnatal day 2–4 mouse brain **(A)** and were identified by immunofluorescent staining with Iba1, CD11b, and GFAP antibody. Purified microglia were shown in **(B)**. GFAP positive astrocytes were found scarcely in the culture while the vast majority of cells were positive for Iba1 and CD11b staining. Unstimulated primary microglia were served as blank for each time point. LPS (10 μg/ml)-stimulated cells (LPS group), nicotine (10 μmol)-treated cells (nicotine group), and LPS (10 μg/ml) plus nicotine (10 μmol)-treated cells (LPS plus nicotine group) were used. Cells were pretreated with LPS 30 min before adding nicotine. Microglia cells incubated with LPS for 24 h were used as the pro-inflammatory control. Supernatant were collected at 12 and 24 h after treatment. Each group had duplicated wells. Pro-inflammatory and anti-inflammatory responses in microglia were evaluated by a commercial cytokine array. Cytokines expression levels were severally determined using a 20-array. Heat map representing cytokine concentrations was shown **(C)**. Quantitative analysis of cytokines secreted from primary microglia were presented in the graph **(D)**. Each group was duplicated, and the mean of the respective cytokine is represented in the heat map and the bar graph.

To further validate the anti-inflammatory effects of nicotine, we used lipopolysaccharide (LPS)-stimulated primary microglia cultures to assess the anti-inflammatory effect of nicotine. Primary microglia were isolated and purified from postnatal day 2–4 mice cerebral cortices (Figure 4A). The purity of primary microglia was identified using immunofluorescent staining, and CD11b + or Iba1+ cells were marked as microglia (Figure 4B). Inflammatory response in microglia was evaluated by commercial cytokine arrays. Cytokine (Figure 4C) expression levels were represented by heat maps. LPS group up-regulated pro-inflammatory factors and down-regulated anti-inflammatory factors compared with blank control. Nicotine attenuated the elevated expression of pro-inflammatory factors (TNFα, IL-6, IL-10, KC, et al.), and the decreased expression of anti-inflammatory factors (IL-4, IL12, IL13, et al.) induced by LPS at 24 h, but had little influence at 12 h. Taken together, it indicates that nicotine exerts anti-inflammatory effects in LPS-induced inflammatory response. To validate the *in vivo* nicotine exposure in mice, we compared nicotine treatment relative to the blank with *in vitro* assay. Concentrations of cytokines (Figure 4D) in the group of nicotine treatment relative to the blank at 24 h were analyzed and shown in the bar graph. Nicotine down-regulated the pro-inflammatory factors and up-regulated the anti-inflammatory factors. These data suggest that nicotine promoted anti-inflammatory response by inhibiting the secretion of pro-inflammatory cytokines in microglia.

### Maternal Nicotine Exposure Skewed the Polarity of Microglia to the M2 Phenotype in Juvenile Offspring

**FIGURE 5:**
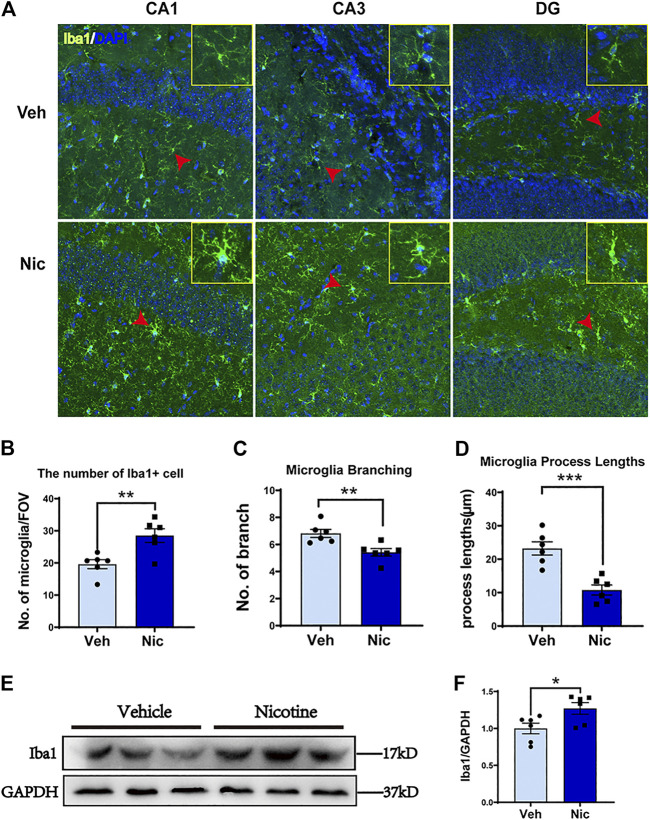
**Nicotine exposure increased the number of Iba1 positive microglial cells and promoted M2-like microglial polarization in the nicotine-exposed offspring’s hippocampus.** The morphology of microglia was detected by immunofluorescence staining. Representative images of the Iba1 expression in CA1, CA3, and DG were shown **(A)**. Olympus FV3000 Confocal Microscopy was used to get a Z-stack for 3-D reconstruction, and images showed that microglia cells changed their morphology from branching (in the control) to amoebic (in the nicotine-exposed group) in the offspring hippocampus. Nicotine exposure increased hippocampus microglial number **(B)**, but decreased microglial branching **(C)** and microglial process lengths **(D)**. CA1, CA3, and DG areas were used to generate the date presented in B-D. A point represented a calculated average of each measurement. **(E, F)** Iba1 protein level was increased in nicotine offspring revealed by western blot, as compared to vehicle offspring. *p < 0.05; **p < 0.01; ***p < 0.001; ****p < 0.0001. Scale bar = 50 μm all data are means ± SEM.

We next investigated whether nicotine promotes the expression of M2 microglia by examining their morphology. The hippocampus Iba1 + staining was shown (Figure 5A). The nicotine offspring presented an increase in the number of hippocampal Iba1+ cells fluorescent staining (*t* = 3.517, *p* = 0.0056, *n* = 6; Figure 5B) as compared to vehicle offspring. The hippocampus microglial cells of the vehicle offspring have a ramified shape with extensively branched processes. The cells of the nicotine offspring developed ameboid morphology characterized by cell body enlargement, decreased cell branching (*t* = 3.46, *p* = 0.0061, *n* = 6; Figure 5C) and shortened cell process lengths (*t* = 5.025, *p* = 0.0005, *n* = 6; Figure 5D). Taken together, these data demonstrate that nicotine promotes M2 microglial morphology in the hippocampus. To further determine the up-expression of hippocampal microglia-specific protein, the microglia-specific Iba1 protein was tested by western blot (Figure 5E). Consistently, an increase in Iba1 protein level was observed in the nicotine offspring (*t* = 2.549, *p* = 0.0289, *n* = 6; Figure 5F) as compared to that in vehicle control mice.

### Nicotine Promoted Expression of BDNF in Hippocampal Microglia

**FIGURE 6:**
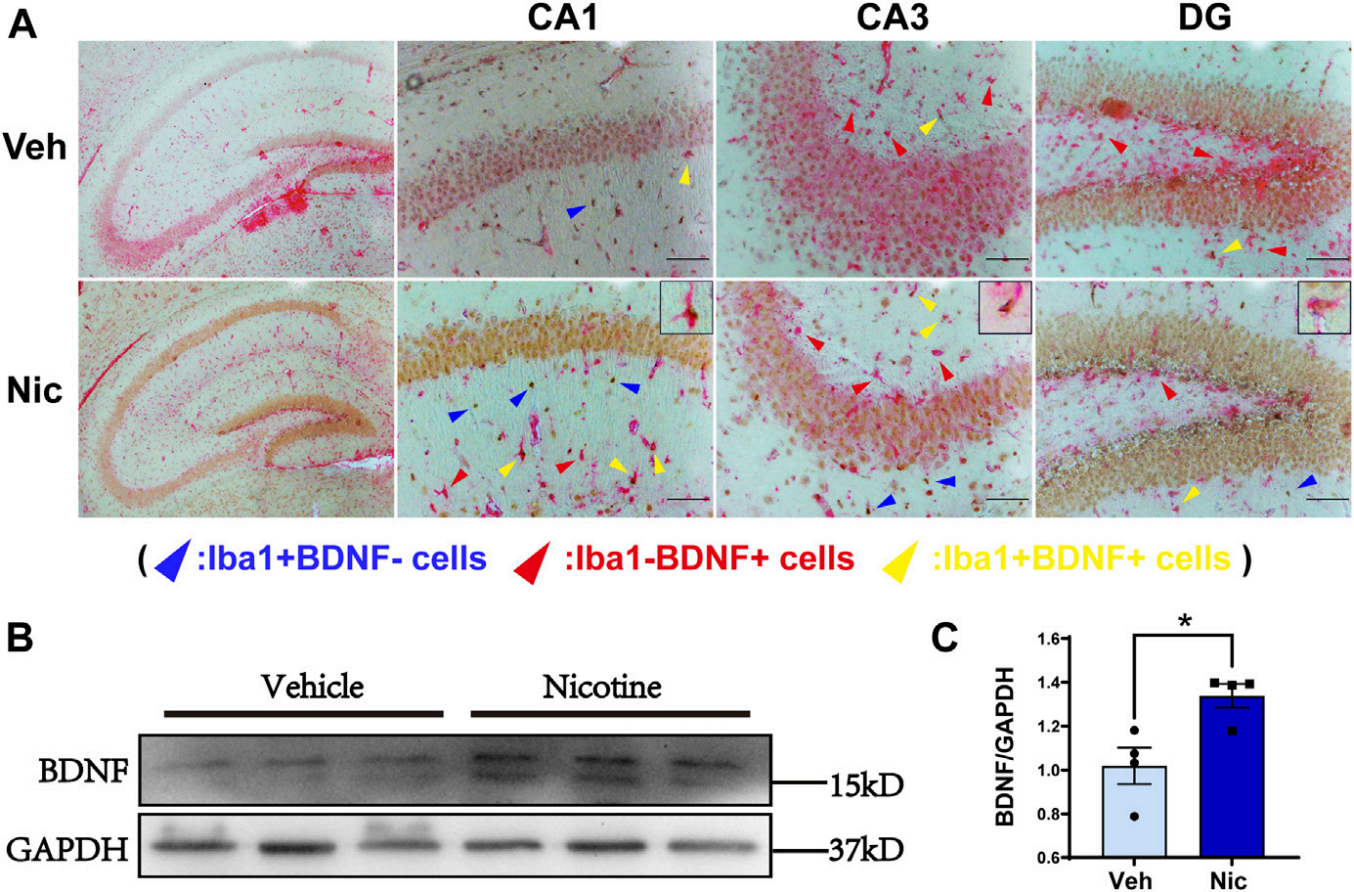
**Maternal nicotine increased the number of microglia Iba1 and BDNF double-positive staining cells.** Low magnification (×100) of the hippocampal coronal sections of vehicle offspring and nicotine offspring (**A**, left) at postnatal day 20, respectively. Iba1-positive cells were marked with DAB (brown) and BDNF-positive cells were marked with AP (red). Increased number of Iba1-positive, BDNF-positive, and Iba1-positive BDNF-positive cells were observed in DG, CA1, CA3. The yellow arrowhead represents Iba1-positive BDNF-positive, the red arrowhead represents Iba1-negative BDNF-positive, and the blue arrowhead represents Iba1-positive BDNF-negative **(A)**. BDNF protein level was distinctly increased in nicotine offspring, as compared to vehicle offspring **(B, C)**. **p < 0.01; Scale bar: 100 μm (A, B); 50 μm (C). all data are means ± SEM.

To further characterize nicotine-induced changes to microglia, we measured the expression of Iba1 and BDNF by immunohistochemical staining. Iba1-positive cells were labeled with DAB (brown), and BDNF-positive cells were labeled with AP (red). There were more Iba1 + cells and BDNF + cells in the nicotine group (Supplementary Figure S2). Then we used double-labeling immunohistochemistry to detect the expression of microglia-derived BDNF. Magnification (×100) of representative the hippocampal coronal sections from the vehicle and nicotine group were shown, respectively, (Figure 6A, left). There were more Iba1+/BDNF− cells (colored with brown, pointed with blue arrowhead) and Iba1−/BDNF + cells (labeled with red, pointed with red arrow) in the hippocampus of the nicotine group. A large fraction of microglial cells were colocalized with BDNF (stained with red and brown) in CA1, CA3, and DG in nicotine offspring as pointed with yellow arrowhead and zoomed in the upper right frame of each image (Figure 6A). These data suggest that nicotine induced an increase in hippocampal microglia, mainly M2 state microglia, characterized by the production of BDNF. To further confirm the upregulation of BDNF, the BDNF protein in hippocampal tissue was tested by western blotting (Figure 6B). Consistently, an increase in BDNF protein level was observed in nicotine offspring (*t* = 3.238, *p* = 0.0177, *n* = 4) as compared to that in vehicle control mice (Figure 6C).

### Maternal Nicotine Exposure Promoted Expressions of Neuronal Markers and Synaptic-Associated Protein in Offspring

**FIGURE 7:**
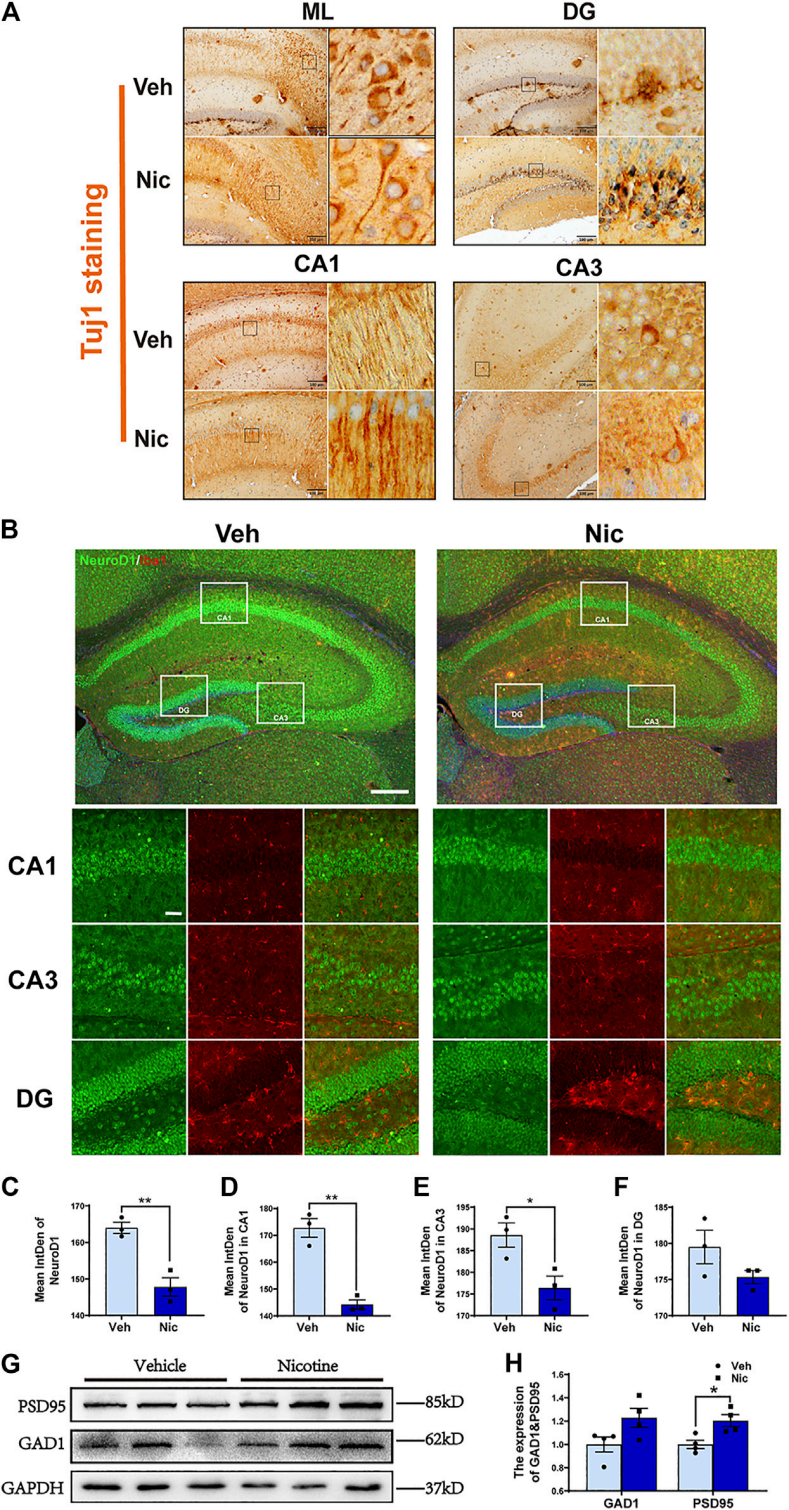
**Maternal nicotine exposure promoted elongation of axons and expression of the synaptic-associated protein in offspring. (A)** Maternal nicotine exposure promoted elongation of axons in ML, DG and CA1, but did not affect on axonal prolongation in CA3. **(B)** NeuroD1-positive cells in the offspring’ hippocampi were analyzed by immunofluorescence staining. Representative images were shown. **(C)** The quantification of fluorescence intensity by ImageJ showed that the immunofluorescence intensity of NeuroD1 decreased obviously in the hippocampus of the nicotine group, including in CA1 **(D)** and CA3 **(E)** not in DG **(F)**. **(G)** Western blot analysis of the activation of PSD95 and GAD1 in the hippocampus of the offspring. **(H)** PSD95 protein level was increased in nicotine offspring as compared to vehicle offspring. There is no difference in GAD1 between nicotine and vehicle treatment. *p < 0.05, **p < 0.01; Scale bar:200 μm (A); 200 μm (low power lens), 50 μm (high power lens) (B). all data are means ± SEM.

To further determine whether nicotine influences neurons, we stained the neuron marker Tuj1 by immunohistochemical staining. Tuj1, neuron-specific class III β-tubulin in the specific differentiation of neuronal cell types was labeled by immunohistochemical staining. One of the notable advantages of using immunohistochemistry to detect Tuj1 is its ability to show axon and terminal detail. Maternal nicotine exposure enhanced elongation of axons in ML (molecular layer), DG and CA1, but had no effect on axonal prolongation in CA3 (Figure 7A). The expression of NeuroD1, a potential regulator for terminal differentiation, neuronal maturation, and survival in the hippocampus, was tested by immunofluorescent staining (Figure 7B). The expression intensity of NeuroD1 positive cells were analyzed using ImageJ. Inversely, the fluorescence intensity of NeuroD1 decreased distinctly in the hippocampus (*t* = 5.536, *p* = 0.0052, *n* = 3) (Figure 7C), mainly including the intensities in CA1 (*t* = 7.367, *p* = 0.0018, *n* = 3) (Figure 7D) and CA3 (*t* = 3.096, *p* = 0.0364, *n* = 3) (Figure 7E), in nicotine group; The control group has greater NeuroD1 intensity in DG (*t* = 1.676, *p* = 0.1691, *n* = 3) compared to the nicotine group, but has no statistically significant difference. (Figure 7F). Then we examined synapse-associated proteins (GAD1 and PSD95) in the hippocampus by western blotting (Figure 7G). An increase in synaptic-associated protein level was observed in both PSD95 (*t* = 3.232, *p* = 0.0179, *n* = 4) and GAD1 (*t* = 2.191, *p* = 0.071, *n* = 4) (Figure 7H) in nicotine offspring as compared to that in vehicle control mice. These data indicated that nicotine induces the synaptic elongation except CA3 region, and promotes the expression of PSD95, there is no statistically significant difference in GAD1 levels between the control and nicotine group, but there is a trend of increase in GAD1 levels in the nicotine group.

## Discussion

Developmental nicotine exposure is associated with much neuronal dysplasia in offspring. Microglia play an important role throughout the development of the brain. However, it is not clear what role microglia plays in the hippocampal impairment caused by developmental nicotine exposure, and what is the effect of nicotine on the differentiation of neural stem cells in the hippocampus. This study is intended to establish a mouse model of maternal nicotine exposure and to explore nicotine-associated impairments on the polarization of hippocampal microglia, neurogenic differentiation, and behavior in juvenile offspring by using a variety of experimental methods. Firstly, our data showed that maternal nicotine exposure leads to offspring neurodevelopment delay and anxiety. Secondly, in the nicotine group, anti-inflammatory factors were up-regulated and proinflammatory factors were down-regulated, which is confirmed by *in vitro* experiment showing that nicotine promotes the secretion of anti-inflammatory cytokines in primary microglia. Thirdly, Maternal nicotine exposure induced the polarization of hippocampal microglia to M2, up-regulated BDNF, increase BDNF + microglia cells, but down-regulated NeuroD1. These data suggest that maternal exposure to nicotine polarized hippocampal microglia toward M2 and secreted BDNF, and microglia *via* anti-inflammatory cytokines and abnormal regulation of hippocampal neural progenitor cell differentiation can influence developmental disorders in offspring.

Nicotine is the major toxic substance in tobacco that causes mental and physical dependence. Oral nicotine administration *via* drinking water has been used in various experiments, such as addiction, withdrawal, and toxicity. The 200 μg/ml nicotine in drinking can produce 100–200 ng/ml blood plasma cotinine, which is equal to the plasma cotinine level of a moderate smoker (Lawson et al., 1998; Mojica et al., 2018). Moreover, the nicotine level in breast milk is even higher than that in the blood, and nicotine in breast milk can be absorbed through the placenta and lactation angiogenesis and cause anxiety-like behavior in offspring (Matta et al., 2007; Karimiankakolaki et al., 2019). Smoking or nicotine exposure during pregnancy is associated with low birth weight (Knopik et al., 2016b; Zeng et al., 2020), which is consistent with the findings of our study. However, during development, the weight gain does not show significant differences between nicotine offspring and vehicle ones. These differences may be a result of fetal tissue hypoxia, intrauterine growth restriction before birth, but the adverse intrauterine environment disappeared without an obvious discrepancy in body weight following birth. Nicotine exposure during pregnancy could lead to adverse effects on neurobehavioral in offspring, such as increased anxiety, depression-like behavior, postnatal hyperactivity, cognitive impairment, severe mental illness disorders et al. (Alkam et al., 2013; Quinn et al., 2017). Similarly, delayed development of eye-opening, auditory startle, and olfactory reflex after birth, as well as anxiety-like behavior presented in the OFT on PND 20 in nicotine juvenile offspring greatly support developmental neurotoxicity of nicotine. Four mice in the vehicle group spent over ∼80% of their time during OFT in the center zone, this might result from individual differences.

We acknowledge that the gender-specific difference in nicotine’s effects on the brain and behavior during the perinatal period has been reported previously in mice (Zhang et al., 2018; Martin et al., 2020). However, when analyzing spontaneous locomotor, hippocampal RNA expression, and neuroinflammation, and the neuronal marker, and focusing on postnatal day 20 when mice were not sexually mature, we did not perform gender-difference analysis upon nicotine’s effect. Firstly because, sex hormones in the male and female hippocampus fluctuated from P0 to P21 (Zuloaga et al., 2014) and increased from as early as embryonic period day 11(E11), and up until postnatal day 21(P21) (Franklin et al., 2019). Secondly, the study of Single-cell RNA Sequencing of microglia revealed nine unique clusters of microglial at three major developmental ages of mice, E14.5, P4/P5, and P100, but does not find any differences in the transcriptomes among males and females mice (Hammond et al., 2019). In this experiment, subjects in each group contain an equal number of males and females, which would not largely impact chronic developmental nicotine exposure on microglia phenotype and function in this study.

To date, microarrays have been extensively used to examine changes in gene expression. Very few studies examine changes of gene expression in the hippocampus induced by nicotine using microarray. We provided the first evidence that a large number of mRNAs related to inflammatory cytokines and chemokines were differentially expressed in developing hippocampus following maternal nicotine exposure. Nicotine juvenile offspring express a different inflammatory-associated transcriptional profile in the hippocampus, as indicated by a decrease in expression of pro-inflammatory chemokines/cytokines and an increased expression of anti-inflammatory chemokines/cytokines. A balance of the pro-and anti-inflammatory response is needed to maintain homeostasis of the CNS. Pro-inflammatory chemokine, such as IFN-γ-induced protein 10 (IP-10, also known as CXCL10), as a classical pro-inflammatory M1 marker (Lescoat et al., 2020), was greatly downregulated following nicotine exposure (Shaykhiev et al., 2009). Anti-inflammatory cytokine IL-4, a multifunctional cytokine, was upregulated in the hippocampus of nicotine offspring and has been reported involved in the regulation of inflammatory responses of the CNS (Zhao et al., 2015). IL-4 in the hippocampus drove M2 microglia to promote neurogenesis, which attenuated depressive-like behaviors (Zhang et al., 2021). Together, these data suggest that nicotine suppresses the M1 response or even tilt the phenotype toward M2.

With chemokines and cytokines alteration, nicotine also alters expression levels of transcripts regulating neurotransmitter release and synaptic function. There are several up-regulated genes within the hippocampus, such as BDNF, NPY2R, and Gria1, and downregulated genes such as Arc, which are well known in the neural plasticity and pathogenesis of central nervous disease and abnormal behavior. In line with this, recent studies also report that increased BDNF expression in the hippocampus is associated with anxiolytic effects (Ma et al., 2017; Onodera et al., 2020). Neurotransmitter receptors on microglia (Lee, 2013) can be activated, and in return modulate pro- and anti-inflammatory function. BDNF, a main member of the neurotrophin family, regulates many neuronal functions including cell differentiation, cell survival, neurotransmission release, and synaptic plasticity. Nicotine exposure enhances the expression of BDNF both *in vivo* and *in vitro* (Lang et al., 2007; Guo et al., 2020). Similarly, BDNF was significantly upregulated in the hippocampus of nicotine offspring, and colocalized with M2-like microglia showed in our data.

Maternal nicotine exposure promotes M2-like polarization of microglia in juvenile offspring. M2-like polarization of microglia cells express and secrete BDNF, attenuate localized inflammation, actively involved in self-tissue repair and coping with adverse intrauterine consequences of offspring after long-term chronic nicotine exposure (Damborsky and Winzer-Serhan, 2012). Moreover, BDNF has been shown to support microglial activation *in vivo* (Jiang et al., 2011), which could lead to microgliosis and amplification of neuroinflammation. Nicotine exposure during pregnancy can impair immune response. Microglia are macrophage-like resident immune cells of the CNS combating infection, clearing cellular debris, and maintaining tissue homeostasis. When the immune system is challenged and microglia are activated, the activated microglia have the potential to function in either a neurodegenerative or a neuroprotective way (Ueno et al., 2013). Some latest investigations suggest that microglia can be activated into a classic activated state (M1 state) or selectively activated state (M2 state) (Ju et al., 2015; Wehrspaun et al., 2015). In the M1 state, activated microglial cells produce proinflammatory cytokines, which are considered to be detrimental; In contrast, activated microglial cells of the M2 state secrete anti-inflammatory cytokines and neurotrophins, including IL-4, BDNF, and glial cell-derived neurotrophic factor (GDNF), which are regarded to be beneficial. The increase in Iba1+/BDNF+ cells within the hippocampus was examined by immunohistochemical staining in our study which confirms that nicotine exposure skews the polarity of microglia to the M2 phenotype, All together these data show that nicotine exposure induces microglia to convert to M2-type cells.

Microglia activation affects both the structural and functional properties of neurons. In response to injury, neurons could produce adhesion molecules and trophic factors that recruit and activate microglial cells and astrocytes (Ramesh et al., 2013). Microglia have been revealed to be active participants in complex neurodevelopmental programs such as neurogenesis and synaptic pruning, in which they interact with neurons and glia to provide nutritional support, respond to cytokine (Cowan and Petri, 2018). Synaptic pruning is a part of the normal developmental process of the brain, occurring at frequent levels in the first weeks after birth. Microglia constantly scan neurons by extending and contracting processes, all the while signaling with neurons and actively maintaining the health of synapses (Zelikoff et al., 2018b). However, the present study and previous studies (Andoh et al., 2020; Iovino et al., 2020) showed that disruption of synaptic pruning by abnormally activated microglia may contribute to cognitive impairment, neurodegenerative diseases, and anxiety-like behavior.

Maternal nicotine exposure affects the maturity and difference of neurons. Neurite outgrowth is a basic process for neurobehavior that involves neuronal differentiation and migration during brain development, which is essential for the communication of the nervous system (Vernes et al., 2011). *In vitro* experiment on brain organic chip model showed that the group treated with low-dose nicotine increased neurite length, suggesting that nicotine at a low concentration triggers a neuronal outgrowth. In contrast, the group treated with high-dose nicotine reduced neurite length, which indicated a disturbed neurite outgrowth (Wang et al., 2018). Tuj1 is a marker of immature neurons, differentiation, and migration, which was revealed to exist in immature neuronal cell bodies, dendrites, axons, and axon terminals (Moon et al., 2019). Likewise, in our study, maternal nicotine exposure increases Tuj1 positive neurite length in ML and CA1. NeuroD1 is principally expressed in the nervous system late in development and is, therefore, more likely to be involved in terminal differentiation, neuronal maturation, and survival (Gaudillière et al., 2004). Furthermore, NeuroD1 was capable of reprogramming neuroglia into functional neurons, which may provide a potential therapeutic approach to restore lost neuronal function in the injured or diseased brain. Therefore, we hypothesized that maternal nicotine exposure was able to stimulate the extension of immature neuronal neurite and over-expression of synapse-associated proteins, but inhibited neuronal maturation and survival by reducing the expression of NeuroD1.

Pharmacological modulation of functional phenotypes of microglia has shown to be a promising cell-based regenerative strategy for neurological diseases (Papa et al., 2016; Song and Suk, 2017). Particular attention is given to utilizing M2 microglial polarization as a potential therapeutic option during the recovery phase (Cherry et al., 2014; Yao and Zu, 2020). For instance, M2-like microglia preserve myelin homeostasis after white matter integrity (WMI) in traumatic brain injury (Wang et al., 2015). M2 microglia obtained by IL-4 stimulation, used for cell transplantation therapy for spinal cord injury, have significantly higher scores for spinal cord injury when compared with systemic administration of IL-4 (Kobashi et al., 2020). IL4-driven microglia phenotypic shift modulated hippocampal neurogenesis and stress in a BDNF-dependent manner (Zhang et al., 2021). The protective mechanisms exerted by M2 microglia to treat CNS injury have also been discussed in recent review articles (Tang and Le, 2016; Du et al., 2017). The treatment polarizing microglia subpopulation toward different phenotypes at different stages of injury might account for future study because microglia polarization has pros and cons (Hu et al., 2015). Based on our finding, M2 microglia-induced therapy in combination with BDNF can be a potential therapeutic strategy for the treatment of neurodevelopmental disorders caused by early life nicotine exposure.

The present study is the first to explore the effects of maternal nicotine exposure on the hippocampal transcriptome and the polarized status of hippocampal microglia in juvenile offspring, as well as the potential roles BDNF playing in the process. The anti-inflammation cytokine and chemokine from microglia induced in the hippocampus of nicotine offspring maybe a kind of protective traumatic stress response. Microglia play a key role in maternal nicotine exposure affecting neurodevelopment in juvenile offspring which would be an interesting study in the future.

## Data Availability

The datasets presented in this study can be found in online repositories. The names of the repository/repositories and accession number(s) can be found in the article/Supplementary Material.
