# Supplementary material for: Maternal Nicotine Exposure Alters Hippocampal Microglia Polarization and Promotes Anti-inflammatory Signaling in Juvenile Offspring in Mice
Source: Front Pharmacol. 2021 May 11;12:661304. doi: 10.3389/fphar.2021.661304 (PMC8144443; doi:10.3389/fphar.2021.661304)
Supplement: Supplementary file 1 [file Table1.DOCX]

**Sample size**

| Vehicle | Body weight | n=44 |
| --- | --- | --- |
|  | Open-field | n=12 |
|  | Nissl staining | n=3 |
|  | Immunofluorescence staining | n=6 |
|  | immunohistochemical staining | n=6 |
|  | Microarray analysis | n=3 |
|  | qPCR | n=3 |
|  | Western Blot | n=6-8 |
| Nicotine | Body weight | n=60 |
|  | Open-field | n=12 |
|  | Nissl staining | n=3 |
|  | Immunofluorescence staining | n=6 |
|  | immunohistochemical staining | n=6 |
|  | Microarray analysis | n=3 |
|  | qPCR | n=3 |
|  | Western Blot | n=6-8 |
