# Supplementary material for: Maternal Nicotine Exposure Alters Hippocampal Microglia Polarization and Promotes Anti-inflammatory Signaling in Juvenile Offspring in Mice
Source: Front Pharmacol. 2021 May 11;12:661304. doi: 10.3389/fphar.2021.661304 (PMC8144443; doi:10.3389/fphar.2021.661304)
Supplement: Supplementary file 2 [file Image1.pdf]

Immunohistochemistry staining

For Iba1 or BDNF staining, slices were incubated with anti-Iba1 or anti-BDNF antibody at 4℃ overnight. After PBST washing, then the sections were incubated with secondary antibodies, donkey anti-goat IgG H&L or donkey anti-rabbit IgG H&L for 40 min at room temperature, and be stained with DAB or AP substrate for 10 min and counterstained with hematoxylin for 30s. Finally, slices were sealed with permanent mounting medium and acquired images.

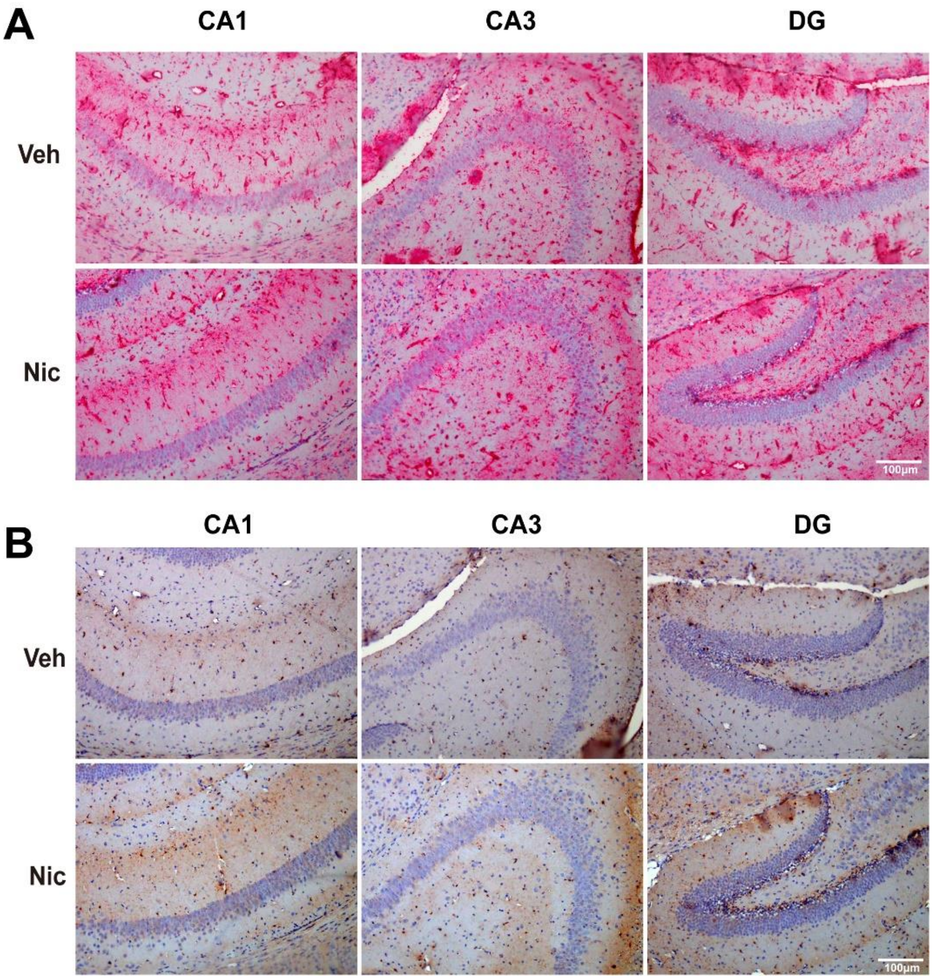

Supplementary Fig. 2: BDNF staining only (A) and Iba1 staining only (B)
